# Supplementary material for: Conditional chemoconnectomics (cCCTomics) as a strategy for efficient and conditional targeting of chemical transmission
Source: eLife. 2024 Apr 30;12:RP91927. doi: 10.7554/eLife.91927 (PMC11060718; doi:10.7554/eLife.91927)
Supplement: Figure 3—source data 3. [file elife-91927-fig3-data3.zip › Figure 3 source data3 part 2.zip!/Figure 3-Source data 3-part2/1060-9-CRISPResso_Report_q0_g4o36.zip!/CRISPResso_on_1060_11/2b.Nucleotide_percentage_quilt_around_sgRNA_gtcctacgtctggtgggtca.pdf]

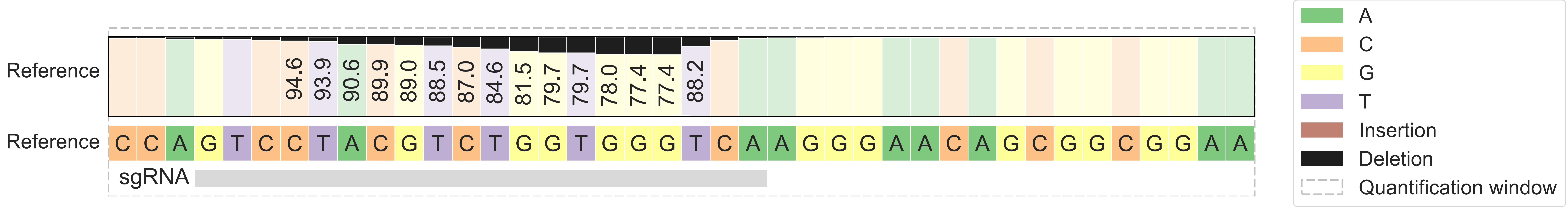

Reference
94.6
93.9
90.6
89.9
89.0
88.5
87.0
84.6
81.5
79.7
79.7
78.0
77.4
77.4
88.2
Reference
C C A G T C C T A C G T C T G G T G G G T C A A G G G A A C A G C G G C G G A A
sgRNA
A
C
G
T
Insertion
Deletion
Quantification window
